# Implementing PROMS for elective surgery patients: feasibility, response rate, degree of recovery and patient acceptability

**DOI:** 10.1186/s41687-022-00483-6

**Published:** 2022-07-07

**Authors:** Natasha K. Brusco, Victoria Atkinson, Jeffrey Woods, Paul S. Myles, Anita Hodge, Cathy Jones, Damien Lloyd, Vincent Rovtar, Amanda M. Clifford, Meg E. Morris

**Affiliations:** 1 Academic and Research Collaborative in Health (ARCH), La Trobe University, Bundoora, VIC Australia; 2 Alpha Crucis Group, Melbourne, VIC Australia; 3 Rehabilitation, Ageing and Independent Living (RAIL) Research Centre, Monash University, Melbourne, VIC Australia; 4 Healthscope Limited, Melbourne, VIC Australia; 5 Anaesthesiology and Perioperative Medicine, Central Clinical School, Alfred Hospital and Monash University, Melbourne, VIC Australia; 6 School of Allied Health, Health Research Institute, Ageing Research Centre, University of Limerick, Limerick, Ireland; 7 Victorian Rehabilitation Centre, Healthscope Limited, Glen Waverley, VIC Australia

**Keywords:** Patient reported outcome measure (PROM), Consumer, Hospital, Feasibility, Acceptability, Co-design, Implementation science, Safety, Quality of recovery

## Abstract

**Background:**

Patient reported outcome measures (PROMs) engage patients in co-evaluation of their health and wellbeing outcomes. This study aimed to determine the feasibility, response rate, degree of recovery and patient acceptability of a PROM survey for elective surgery.

**Methods:**

We sampled patients with a broad range of elective surgeries from four major Australian hospitals to evaluate (1) feasibility of the technology used to implement the PROMs across geographically dispersed sites, (2) response rates for automated short message service (SMS) versus email survey delivery formats, (3) the degree of recovery at one and four weeks post-surgery as measured by the Quality of Recovery 15 Item PROM (QoR-15), and (4) patient acceptability of PROMS based on survey and focus group results. Feasibility and acceptability recommendations were then co-designed with stakeholders, based on the data.

**Results:**

Over three months there were 5985 surveys responses from 20,052 surveys (30% response rate). Feasibility testing revealed minor and infrequent technical difficulties in automated email and SMS administration of PROMs prior to surgery. The response rate for the QoR-15 was 34.8% (n = 3108/8919) for SMS and 25.8% (n = 2877/11,133) for email. Mean QoR-15 scores were 122.1 (SD 25.2; n = 1021); 113.1 (SD 27.7; n = 1906) and 123.4 (SD 26.84; n = 1051) for pre-surgery and one and four weeks post-surgery, respectively. One week after surgery, 825 of the 1906 responses (43%) exceeded 122.6 (pre-surgery average), and at four weeks post-surgery, 676 of the 1051 responses (64%) exceeded 122.6 (pre-surgery average). The PROM survey was highly acceptable with 76% (n = 2830/3739) of patients rating 8/10 or above for acceptability. Fourteen patient driven recommendations were then co-developed.

**Conclusion:**

Administering PROMS electronically for elective surgery hospital patients was feasible, acceptable and discriminated changes in surgical recovery over time. Patient co-design and involvement provided innovative and practical solutions to implementation and new recommendations for implementation.

*Trial Registration and Ethical Approval* ACTRN12621000298819 (Phase I and II) and ACTRN12621000969864 (Phase III). Ethics approval has been obtained from La Trobe University (Australia) Human Research Ethics Committee (HEC20479).

**Key points:**

Patient reported outcome measures (PROMs) help to engage patients in understanding their health and wellbeing outcomes. This study aimed to determine how patients feel about completing a PROM survey before and after elective surgery, and to develop a set of recommendations on how to roll out the survey, based on patient feedback. We found that implementing an electronic PROM survey before and after elective surgery was relatively easy to do and was well accepted by patients. Consumer feedback throughout the project enabled co-design of innovative and practical solutions to PROM survey administration.

## Introduction

Patient reported outcome measures (PROMS) provide information on patient perceptions of their own health and wellbeing, including in response to interventions such as elective surgery [1–3]. By engaging patients in co-evaluation of their responses to surgery, health professionals and administrators can implement patient-informed, co-produced policies, procedures and interventions [4, 5]. Prior to large scale implementation of PROMS across hospitals globally, it is important to understand PROM feasibility, acceptability and outcomes from a surgical patient perspective [6, 7].

Feasibility refers to the ease of treatment implementation, practicality, integration, demand and acceptability [8]. In the context of receiving a PROM survey, each of the constructs are key. For example, the burden of a survey refers to the effort required to participate at different time points, coherence is the extent to which the patient understands the survey, and the self-efficacy refers to patient confidence that they can complete the survey correctly [9]. Acceptability from the patient perspective is particularly important for PROM surveys, as patient response rate, a key outcome of PROM implementation, acts as a surrogate for acceptability [10]. Acceptability is described as the extent to which the people who provide or receive an intervention view it as appropriate, effective and helpful [9]. It also incorporates elements of burden, coherence, ethics, opportunity cost and self-efficacy [9].

This current PROM study for elective surgery patients is a part of a larger research program designed to develop Australian ePROM implementation recommendations, called ‘AusPROM’, with a detailed protocol previously published [11]. In summary, the AusPROM research program contains a number of phased studies that embed patient and staff co-design into the implementation process, through an iterative process, and this included the identification of barriers and facilitators from the patient and staff perspective.

In the current study we sampled patients having a broad range of elective surgeries from four major Australian hospitals, aiming to evaluate (1) technical feasibility of the technology used to implement the PROMs across geographically dispersed sites, (2) response rates for automated short message service (SMS) versus email survey delivery, (3) the degree of recovery at one and four weeks post-surgery (Quality of Recovery 15 Item PROM; QoR-15) [12, 13], and (4) patient acceptability based on survey and focus group results. The patient surveys and focus group were designed to enable patient co-design in the development of the broader AusPROM recommendations, through the development of a set of patient-driven recommendations.

## Methods

This study has been reported according to the CONSORT statement: extension to randomised pilot and feasibility trials [14] in “Appendix 1”. As noted in the aims, the current study reports on four distinct areas from this broader research program including technical feasibility, response rate, degree of recovery and patient acceptability.

### Study design

We used a mixed-methods design to test the technical feasibility, response rate, degree of recovery and patient acceptability of electronic PROMS.

### Patient co-design

To improve acceptability, usability and uptake, patient feedback and co-design were embedded throughout the study [4]. This included a patient co-designing and co-authoring the project from its concept (VR); patients completing acceptability questions alongside the PROM survey; as well as a patient focus group to further explore PROM survey acceptability and patient driven recommendations for ongoing PROM implementation [4, 5].

### Participants

Survey participants were patients who had elective surgery in four hospitals in Australia. Inclusion criteria were adults aged 18+, having either elective day-surgery or elective surgery requiring an overnight hospital admission. Exclusion criteria included a pregnancy related procedure or an investigative procedure (see “Appendix 2” for full list of excluded procedures). Consent was via an opt-in consent tick-box prior to commencing the survey. The pre-test surveys were distributed through one hospital in New South Wales, Australia. The pilot site surveys were distributed through four of the health services 29 hospitals which provide elective surgery. The four pilot sites were selected based on a sample of convenience and were located in New South Wales, Queensland and Victoria, Australia. Three of the four hospitals had an emergency department and all four were located in metropolitan areas.

Across the health service, there are hospital specific consumer groups. The consumers are generally past patients from the health service. The patient focus group participants were members of the hospital specific consumer groups (i.e., not current patients). Individuals were approached through the Quality Manager, based on a request from the Corporate Consumer Consultant (Chairperson of the Healthscope National Consumer Advisory Council). The sample of convenience aimed to obtain a mix of past patients across the different states and territories of Australia. Inclusion criteria were adults 18+ who were members of the hospital specific consumer groups. There were no exclusion criteria. Written informed consent was required prior to participation in the focus group.

### Intervention and study instrument

The intervention was the administration of the PROM survey, based on the QoR-15 tool, the week prior to surgery, and one and four weeks post-surgery. The QoR-15 was derived from the Quality of Recovery 40 item (QoR-40) tool [12]. It has 15 items each rated on a 11-point scale from 0 to 10, with a maximum score of 150 The QoR-15 has reported good validity, reliability and responsiveness and is brief to administer (< 3 min) [12, 15]. The QoR-15 can be used pre-surgery, 24 h post-surgery, as well as from weeks to months post-surgery, as a measure of change over time [13, 16, 17], supporting this studies use of the QoR-15 the week prior to surgery, and one and four weeks post-surgery. The minimal clinical important difference of the QoR-15 is 8.0 [16].

With permission from the QoR-15 author [13], questions 7 and 8 of the QoR-15 tool were modified for the pre-surgery surveys as they were designed for the post-surgery period. Questions 7 was modified from “Getting support from hospital doctors and nurses” to “Getting support from Health Professionals”, and Question 8 was modified from “Able to return to work or usual home activities” to “Able to participate in work or usual home activities”.

The QoR-15 tool was chosen due to its valid use pre and post-surgery, the short completion time and its applicability across most surgery types [13, 18–20], enabling the health service to implement one consistent and inclusive tool. In addition, the individual items within the tool can be used to isolate the more difficult areas of recovery post-surgery, and enable the health service to target these areas to improve care and recovery.

### Outcomes

(i) Technical feasibility of the technology used to implement the PROMs across geographically dispersed sites

Outputs and outcomes for the PROM pre-pilot surveys included development of agreed list of surgical procedures to be excluded from the survey distribution list, as well as observations of the differences between the pre-surgery and the post-surgery survey distribution lists.

(ii) Response rates for automated short message service (SMS) versus email survey delivery

Response rate for SMS and email survey invitations were reported separately and combined for the pre-surgery, one week post-surgery and four weeks post-surgery surveys. In addition, at the four sites, separate to the PROM survey, was a long-standing Patient Experience survey. As a part of PROM feasibility testing, we examined if the introduction of the PROM survey impacted the response rate of the Patient Experience survey. To test this, the Patient Experience response rates were reviewed across the four sites during the PROM study.

(iii) Degree of recovery at one and four weeks post-surgery as measured by the QoR-15

The PROM survey, focussed on the QoR-15, was administered electronically pre-surgery, one week post-surgery and four weeks post-surgery. Patients were independently invited to participate in a survey at each time point. This was because someone included in the pre-surgery survey may have been excluded in the post-surgery survey if the planned surgery changed and was one of the excluded surgery types.

(iv) Patient acceptability based on survey and focus group results (quantitative and qualitative acceptability questions)

Outcomes for patient acceptability, via survey questions and the patient focus group, include a survey question “Based on the different aspects of acceptability which are important to you, how do you rate the acceptability of the survey just completed?” measuring of acceptability based on a 0–10 Likert scale (0 = Not acceptable, 10 = Highly acceptable), and an open-ended question asking “Can you please note which aspects of acceptability are important to you? And, how we could modify the survey to be more acceptable to you (or if it is ok just the way it is)?”. In addition, there was a focus group for further qualitative data on acceptability.

The patient survey and focus group results were used to help develop patient driven recommendations for ongoing PROM implementation. It is noted that this study only reports on the patient perspective and that similar work was completed for the staff perspective and this has been reported separately.

The theoretical framework chosen to support the selection and implementation of PROMs was the “PROM cycle” [21]. The PROM cycle has four main phases [21]. The first is “goal” setting, to determine the objective for PROM implementation; the second phase is “selection” and testing of the appropriate PROM; the third identifies the “indicator” which includes the steps of defining and testing the quality indicator; and the fourth is “use” and involves the steps of implementing and evaluating the PROM [21]. Feasibility studies such as the current investigation are vitally important during phases two and three of the PROM-cycle, to test the PROM and confirm quality indicators to show successful implementation.

### Sample size

The PROM pre-pilot surveys aimed to invite up to 100 participants, the survey implementation at four pilot sites aimed to invite up to 2700 participants, and both were based on a sample of convenience. Based on a previous national survey response rate across the same heath service of 37% [22], the PROM survey response rate was also expected to be between 30–40%. The patient focus group aimed for up to 10 participants, and was conducted after the survey results were analysed to ensure the themes which emerged in the survey, were explored in the focus group.

### Statistical analysis

Results from the PROM pre-pilot surveys are reported descriptively. Results for the PROM survey implementation at four pilot sites are reported as a number and percentage for the response rate, and as a mean and standard deviation for the QoR-15 scores pre and post-surgery. Pre-surgery QoR-15 scores were compared to post-surgery scores using independent t-tests reporting the mean difference and confidence interval. A chi-square statistic was used to determine if there was a difference between proportion within age groups, proportion female, and proportion of day surgery versus overnight surgery between pre-surgery, one week post-surgery and four week post-surgery groups. Responses are reported separately for day surgery, overnight surgery and for combined day/overnight surgery, as a number of the surgeons perform surgery which requires both day and overnight admissions, and this reporting structure will ensure aggregate results are meaningful to the surgeons. Statistical significance was assumed at *p* < 0.05 and SPSS [23] was used for the analysis.

Qualitative results for patient acceptability, via survey questions and the patient focus group, were analysed using a content analysis and then themed according to the seven constructs of the theoretical framework of acceptability (TFA) [9]. The constructs were based on affective attitude: surgical patient feelings about the survey; burden: the effort required by the patient to participate; and perceived effectiveness: perception by the patient that the survey is likely to achieve its purpose. In addition there was ethicality: the surveys “fit” with individual value systems; intervention coherence: extent to which the patient understands the survey; opportunity costs: extent to which benefits or values of the patient must be given up to engage in the survey; and self-efficacy: patients confidence that they can complete the survey correctly [9]. These themed qualitative results were then critically reviewed by the research team to derive feasibility and acceptability recommendations from the patient perspective.

## Results

(i) Technical feasibility of the technology used to implement the PROMs across geographically dispersed sites

In April 2021, the pre-pilot survey used email to invite 80 patients to participate over the pre-pilot period. While all 80 received the pre-surgery survey, only 67 received the post-surgery surveys. Patient survey distribution lists included for the pre-surgery survey and the post-surgery survey differed because of late additions or cancellations prior to surgery, and intra-operative changes to the planned surgery, for example a patients would be excluded from the pre-surgery PROM survey distribution list if they had an investigative procedure planned, however, if this extended to an interventional procedure, they would be added to the post-surgery survey distribution list. A list of surgical procedures which were excluded has been detailed in “Appendix 2”.

The pre-surgery survey had 26 responses (response rate 32.5%, n = 26/80). The one and four weeks post-surgery surveys each had 15 responses (response rate 22.4%, n = 15/67). The technical feasibility testing on the pre-pilot survey resulted in a robust party automated/party manual process being developed for the identification of the pre-surgery PROM survey distribution list for PROM survey implementation at four pilot sites, compared to the fully automated post-surgery PROM survey distribution list.

(ii) Response rates for automated short message service (SMS) versus email survey delivery

Between April 2021 and July 2021, there were 5985 surveys responses (response rate 29.8%, n = 5985/20,052) and these were distributed across the pre-surgery survey (response rate 44.5%, n = 1756/3944), the one-week post-surgery survey (response rate 33.3%, n = 2682/8054) and the four weeks post-surgery survey (response rate 19.2%, n = 1547/8054).

Two sites used SMS and two sites used email to invite patients to participate. The response rate for SMS was 34.8% (n = 3108/8919) and the median time to complete the survey was 2 min and 10 s and the response rate for email was 25.8% (n = 2877/11,133) and the median time to complete the survey was 2 min and 29 s (Table 1).

**Table 1:** Response rate

Email versus SMS		Pre-surgery	1 Week post-surgery	4 Weeks post-surgery	Overall
Email	Sent	2039	4547	4547	11,133
	Responses	724	1373	780	2877
	Response rate	35.5%	30.2%	17.2%	25.8%
Short message service (SMS)	Sent	1905	3507	3507	8919
	Responses	1032	1309	767	3108
	Response rate	54.2%	37.3%	21.9%	34.8%
Overall		Pre-surgery	1 Week post-surgery	4 Weeks post-surgery	Overall
	Sent	3944	8054	8054	20,052
	Responses	1756	2682	1547	5985
	Response rate	44.5%	33.3%	19.2%	29.8%

At the four sites, separate to the PROM survey, was a long standing Patient Experience survey. As a part of PROM feasibility testing, we examined if the introduction of the PROM survey impacted the response rate of the Patient Experience survey. To test this, the Patient Experience response rates were reviewed and these remained consistent across the four sites during the PROM study with a response rate ranging from 29.9 to 31.5% prior to introducing the PROM survey (October to December 202), and a rate ranging from 29.4 to 31.7% during PROM survey introduction (April to June 2021; Table 2).

**Table 2:** PROM survey impact on the previously implemented Patient Experience questionnaire

	Oct to Dec 2020 (%)	Jan to March 2021 (%)	April to June 2021 (%)
Hospital A	31.3	31.9	30.0
Hospital B	31.5	30.9	31.7
Hospital C	29.9	30.8	29.4
Hospital D	31.4	30.9	31.5

While patient characteristics were similar for those who completed the pre-surgery survey, compared to the one week post-surgery survey, compared to the four weeks post-surgery, based on a chi-square statistic the proportion of patients in each of the age brackets did significantly differ between the groups (*p* < 0.01), however the difference between proportion female, and proportion of day surgery versus overnight surgery, did not significantly differ (*p* > 0.05), (Table 3).

(iii) Degree of recovery at one and four weeks post-surgery as measured by the Quality of Recovery 15 Item PROM (QoR-15)

**Table 3:** Patient characteristics for the pre-surgery survey, and the one and four weeks post-surgery surveys

	Pre-surgery n = 1756	1 Week post-surgery n = 2682	4 Weeks post-surgery n = 1547
Age range	18–40, 486 (27.7%) 41–64, 763 (43.5%) 65–74, 327 (18.6%) 75–84, 152 (8.7%) 85+, 28 (1.6%)	18–40, 682 (25.4%) 41–64, 1168 (43.5%) 65–74, 547 (20.4%) 75–84, 241 (9.0%) 85+, 44 (1.6%)	18–40, 287 (18.6%) 41–64, 685 (44.3%) 65–74, 371 (24.0%) 75–84, 183 (11.8%) 85+, 21 (1.4%)
Gender, female	1047 (59.6%)	1599 (59.7%)	881 (57.0%)
Day surgery	354 (51.8%)	1255 (47.2%)	720 (46.9%)
Admitted overnight surgery	329 (48.2%)	1402 (52.8%)	815 (53.1%)

Across the four pilot sites there were 489 different surgeons who had patients complete a PROM survey, with each surgeon having an average of 3.50 (SD 5.68) patients complete the pre-surgery survey, 6.44 (SD 7.54) patients complete the 1 week post-surgery survey and 4.32 (SD 4.50) patients complete the 4 weeks post-surgery survey.

**Fig. 1:**
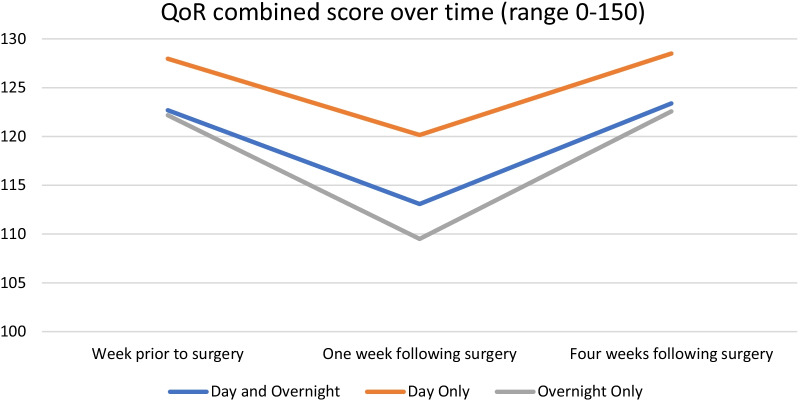
Overall PROM response on the QoR-15 prior to surgery and one and four weeks following surgery

For combined overnight and day admissions, prior to surgery, the mean QoR-15 score was 122.69 (SD 25.23; n = 1021), one week post-surgery it was 113.08 (SD 27.74; n = 1906) and 4 weeks post-surgery it was 123.39 (SD 26.84; n = 1051). While there was a significant difference in the score from pre-surgery to one week following surgery, and again from one and four weeks post-surgery, there was no significant difference between QoR-15 scores pre-surgery and four weeks post-surgery. At one-week post-surgery, 825 of the 1,906 responses (43%) exceeded 122.6 (pre-surgery average), and at four weeks post-surgery, 676 of the 1,051 responses (64%) exceeded 122.6 (pre-surgery average). When this was separated into the subgroups of day admissions and overnight admissions, the findings were similar (Table 4; Fig. 1).

(iv) Patient acceptability based on survey and focus group results (quantitative and qualitative acceptability questions)

**Table 4:** QoR-15 scores

	Pre-surgery	1 Week post-surgery	4 Weeks post-surgery	Mean difference (95% CI, *p* value)		
				1 Week post-surgery minus pre-surgery	4 Weeks post-surgery minus 1 week post-surgery	4 Weeks post-surgery minus pre-surgery
Combined overnight and day admissions	122.69 (SD 25.23; n = 1021)	113.08 (SD 27.74; n = 1906)	123.39 (SD 26.84; n = 1051)	− 9.62 (− 11.66 to − 7.57; *p* < 0.000)	10.31 (8.25 to 12.38; *p* < 0.000)	0.69 (− 1.55 to 2.94; *p* = 0.545)
Overnight stay admissions	122.18 (SD 22.77; n = 514)	109.50 (SD 25.71; n = 968)	122.77 (SD 24.40; n = 551)	− 12.68 (− 15.33 to − 10.03; *p* < 0.000)	13.28 (10.63 to 15.92; *p* < 0.000)	0.60 (− 2.25 to 3.44; *p* = 0.680)
Day admissions	127.97 (SD 19.72; n = 400)	120.15 (SD 23.53; n = 808)	128.50 (SD 22.50; n = 418)	− 7.81 (− 10.49 to − 5.13; *p* < 0.000)	8.35 (5.61 to 11.09; *p* < 0.000)	0.54 (− 2.37 to 3.45; *p* = 0.717)

**Fig. 2:**
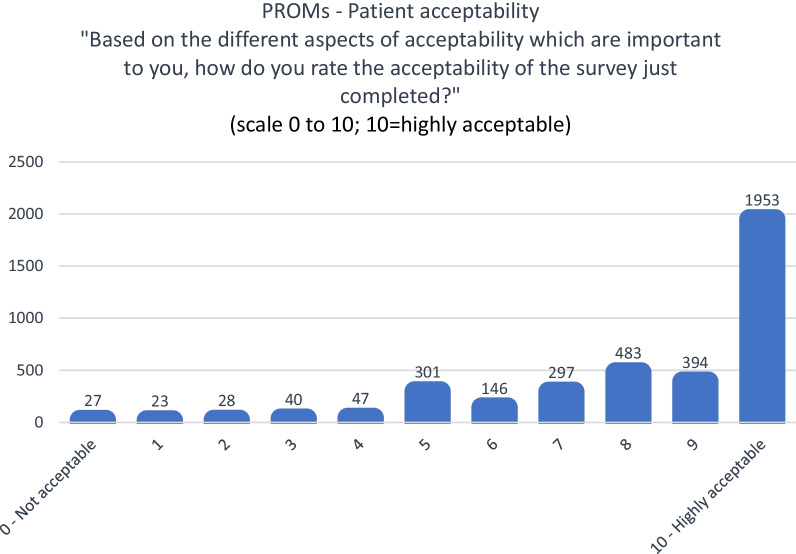
Patient rating for PROM survey acceptability

During the first, second and third surveys, there were 3739 responses to the survey question rating acceptability, 76% (n = 2830/3739) rated 8/10 or above for acceptability where 10/10 indicated highly acceptable (Fig. 2).

In the PROM survey, 1877 (31%) patients provided a breadth of additional comments regarding patient acceptability and 4108 (69%) did not. Comments were spread across the pre-surgery surgery (n = 507, 27%), the one week post-surgery survey (n = 863, 46%) and the four week post-surgery survey (n = 507, 27%). The single patient focus group (n = 8 participants) had representation from NT, SA, NSW, ACT and Victoria, and explored patient acceptability of introducing a PROM survey (focus group interview guide; “Appendix 3”).

### COVID-19 response during pilot data collection

It is noted that the COVID-19 pandemic had an impact on the study during the final weeks of data collection. This involved stopping the pre-surgery survey at two sites (New South Wales) two weeks earlier than planned (completing 10 weeks of the 12 weeks of planned data collection), due to the manual steps associated with the pre-surgery survey to remove participants with excluded surgery types. This was defined as a project “Red Phase”—refer to “Appendix 4” for details related to the “*COVID-19 contingency plan*”. The one week and four weeks post-surgery surveys were not impacted.

### Feasibility and acceptability patient recommendations

To provide structure, the patient recommendations have been presented under the 4-Phases of the PROM cycle as well as the 7-domains of patient acceptability for receiving a health service, in the following Text Box.

TEXT BOX: Patient recommendations *4-Phases of the PROM cycle*

**Phase 1:** Goal setting, to determine the objective for PROM implementation

Recommendation 1: The objective for PROM implementation is to embed PROMs and patient-centred care into the organisations culture. Therefore, as a part of the implementation plan, strategies are put in place to build awareness of PROMs across all hospital staff from the ground up.

**Phase 2:** Selection and testing of the appropriate PROM

Recommendation 2: As the pre-surgery survey distribution is technically difficult due to the high degree of manual input required (not fully automated), it is recommended that in the next Phase of the trial (national PROM survey implementation across the 29 hospitals), we test the patients ability to accurately recall pre-surgery status by adding an extra question to this effect, to the post-surgery surveys. If recall can be achieved with a reasonable degree of accuracy, consider removing the pre-surgery survey.

Recommendation 3: Develop a contingency plan for PROM survey distribution for hospitals responding to a state of emergency (e.g., COVID-19), especially in relation to the pre-surgery survey distribution due to the degree of manual input required.

**Phase 3:** Identify the indicators, which includes defining and testing the quality indicator

Recommendation 4: As a higher survey response rate was achieved through SMS survey distribution compared to email survey distribution, continue to use SMS as the preferred method of survey distribution.

Recommendation 5: Develop a set of PROM Key Performance Indicators (KPIs) which are reported at the department, hospital and executive level.

**Phase 4:** The PROM use, which includes implementing and evaluating the PROM

Recommendation 5 *repeated*: (*evaluating the PROM*) Develop a set of PROM KPIs which are reported at the department, hospital and executive level.

Recommendation 6: (*implementing the PROM*) Doctors and nurses could explain benefits, the importance, and the significance of PROMs to their patients prior to surgery during pre-operative visits and via the hospital pre-admission clinic to support uptake.

Recommendation 7: (*implementing the PROM*) While clinical staff, such as nurses, are encouraged to discuss PROMs with the patients, they are not responsible for administering the PROM survey. Administering the PROM survey should be the responsibility of non-clinical staff such as a data manager who can distribute electronic surveys to the patients.

*7-Domains of patient acceptability*

**Affective attitude**, how the patients feel about the survey

Recommendation 8: A small number of patients (n = 24/5,985; 0.4%) provided feedback relating to mental health. The feedback ranged from describing how they were feeling to applauding the survey for including questions on mental health. It is recommended that at the end of the survey there is a statement to the following effect “If this has brought up feelings of concern, we suggest you contact your health care professional.”

**Burden**, refers to the effort required to participate

Recommendation 9: To minimise the burden, reduce survey fatigue and improve the completion rate, the ideal timing of the surveys has been explored. However, feedback from patients did not provide consistency. It is therefore recommended that in addition to the structured PROM surveys sent out by the hospital, patients are able to access the same PROM survey at any time, via the hospital website, with results emailed directly to the patient. This will ensure the patient can re-visit the PROM survey as often as they like, at the time that they like, to track their own progress.

**Perceived effectiveness**, perception that the survey is likely to achieve its purpose

Recommendation 10: If patients feel that there may be personal benefit in completing their PROMs surveys, rather than the survey being for the sole purpose of Healthscope’s organisational goals, there may be an increase in participation. This would involve the individual survey results going back to the doctor and / or patient once the survey is completed. The results could then be discussed at the next follow up appointment.

**Ethicality**, the surveys “fit” with an individual’s value system

Recommendation 11: To ensure the PROM survey aligns to the patients values, in addition to the emailed/SMS invitation to participate in PROMs, there could be a link to an information on the Healthscope website (in easy-read English and other languages), explaining the purpose, goals and benefits of PROMs for both the patient and for the organisation.

**Intervention coherence**, extent to which the patient understands the survey

Recommendation 12: Concern was raised by patients for survey participants who fall within an older age group, due to a perception that they may struggle to complete their PROMs survey online. However, across the four pilot sites around a third of responses were from patients aged 65 and above. It is therefore recommended that there is ongoing monitoring of the PROM survey response rate across the different age groups to ensure appropriate age group representation. If there is a gap in appropriate age group representation, other modes of PROM survey distribution can be considered.

**Opportunity costs**, extent to which benefits or values must be given up to engage in the survey

Recommendation 13: The extent to which other patient specific benefits / activities were given up to engage in the survey remains unknown. However, through careful monitoring of established hospital patient survey response rates, it was found that introducing a new PROM survey had no opportunity cost to the response rate of the organisations established patient surveys. It is recommended that the response rate to established hospital patient surveys continues to be monitored.

**Self-efficacy,** patients’ confidence that they can complete the survey correctly

Recommendation 14: The vast majority of patients reported that the survey was clear and easy to use. However, a small number of patents noted that one point the scale within the PROM survey changes from a 0–10 rating to a 10–0 rating. It is recommended that buttons for 10 to 0 are shaded, with the 0 to 10 shaded in reverse, to ensure patients see the reversal of the scale.

## Discusssion

This authorship team has completed a research program which resulted in the development of Australian ePROM implementation recommendations, called ‘AusPROM’ [11, 24]. The current study represents the consumer voice and brings patient co-design into the AusPROM recommendation development process. Patients articulated how they felt about completing a PROM survey before and after elective surgery, and co-designed a set of “patient driven” recommendations on how to roll out the survey. These “patient driven” recommendations provided innovative and practical solutions regarding how the survey could be rolled out. These recommendations have been a key driver in a Delphi technique that was used to confirm the final set of AusPROM recommendations [24].

The current study found that implementing PROMS electronically for elective surgery hospital patients was technically feasible and did not require additional infrastructure. In agreement with a 2022 systematic review of global studies by Sokas [25] hospital patients found participating in the ePROM acceptable before and after their surgery, and achieved a similar response rate to other long term hospital surveys. Based on the general direction of the results, for this elective surgery group of patients the PROM discriminated change in the patients surgical recovery over time, with a full recovery for most patients by four-weeks.

Despite being conducted in the height of the COVID-19 pandemic, this comprehensive analysis had almost 6,000 survey responses. It showed favourable responses for implementing PROMS, which is consistent with PROMS implementation in other diagnostic categories such oncology [26] and total knee replacement [27], as well as across broader patient populations [25, 28]. Feasibility testing revealed minor and infrequent technical difficulties in automated SMS or email administration of the PROM prior to surgery. The QoR-15 response rate was higher for SMS compared to email. From the week prior to surgery to the week immediately after elective surgery, patients experienced reduction in this patient-centred outcome and this was reversed by four weeks post-surgery. From the patient perspective, the PROM survey was highly acceptable with 76% of patients rating 8/10 or above for acceptability.

When considering all online devices, such as smart phones and computers, email invitations and SMS invitations to survey participation have a similar response rate, however, when only considering the smart phone response rate, the SMS yields a higher response rate [29, 30]. This finding was consistent for the current study where the SMS response rate (36%) exceeded the email response rate (25%).

The average quality of recovery score in the current study, measured through the QoR-15 tool, prior to elective surgery was 123 out of a possible 150 (n = 1021). This pre-surgery score was consistent with other patient cohorts undergoing mixed surgery types with similar pre-surgery scores of 123 (n = 127) [12] and 125 (n = 363) [31], yet higher than a patient cohort undergoing hip replacement with a score of 114 (n = 115) [32]. Post-operatively, the average quality of recovery score in the current study dipped to 113 (n = 1906) the week after surgery then returned to 123 (n = 1051) 4 weeks after surgery, indicating that based on the general direction of the results, the PROM discriminated change in the patients surgical recovery over time, consistent with the literature [12, 31]. When the cohort was separated into the subgroups of day admissions and overnight admissions, the findings were similar. It is possible that this data set does not represent the final status of recovery after surgery, as further gains may have been achieved after the 4 week post-surgery time point.

Patient acceptability of the current PROM survey was high, based on quantitative and qualitative acceptability questions within the survey and a patient focus group. In comparison, a small number of systematic reviews have concluded that there is either some evidence that PROMs are acceptable to the patients affected by cancer or cystic fibrosis [33, 34], or that there is a paucity in the literature to conclude that PROMs are (or are not) acceptable to the patients affected by chronic fatigue syndrome, a hip fracture or kidney disease [35–37].

It has been described as *wrong* to seek patient feedback in healthcare and then not use this information to influence clinical practice [38]. From the onset, the current study intended to use patient feedback to guide innovative and practical solutions to shape co-design. The themed qualitative results, which developed the 14 feasibility and acceptability recommendations from the patient perspective, will provide a patient voice to the next stage of this research program where the final “AusPROM” Recommendations [11, 24] will be developed. Patient recommendations included defined roles and responsibilities for the nursing, medical, administrative and management staff in relation to the PROM survey process. However, it is unknown if these views would have differed prior to the impact on staff demands due to the COVID-19 pandemic.

Limitations to this study included an end point of 4 week post-surgery which may not have reported the final status of recovery after surgery, excluding any non-electronic modes of survey distribution, as well as the impact of COVID-19 during the final weeks of data collection. The study was conducted in Australia and it is not known whether similar results would be obtained in other regions of the world or for different cultures or case-mixes, or in health services with different technology systems.

## Conclusion

Implementing PROMS electronically for elective surgery hospital patients was feasible, acceptable and showed changes in outcome over time. Patient involvement facilitated innovative and practical solutions to implementation and the formulation of recommendations.

## Data Availability

The named authors on this protocol will have access to the final trial dataset. Individual patient level data will not be available for sharing at the conclusion of this study.

## Appendix 1

### CONSORT statement: extension to randomised pilot and feasibility trials

**Table:**

Section/topic and item no	Extension for pilot trials	Note where reported
Title and abstract		
1a	Identification as a pilot or feasibility randomised trial in the title	✓ Title
1b	Structured summary of pilot trial design, methods, results, and conclusions (for specific guidance see CONSORT abstract extension for pilot trials)	✓ Abstract
Introduction		
Background and objectives:		
2a	Scientific background and explanation of rationale for future definitive trial, and reasons for randomised pilot trial	✓ Introduction
2b	Specific objectives or research questions for pilot trial	✓ Introduction
Methods		
Trial design:		
3a	Description of pilot trial design (such as parallel, factorial) including allocation ratio	✓ Methods
3b	Important changes to methods after pilot trial commencement (such as eligibility criteria), with reasons	✓ Methods
Participants:		
4a	Eligibility criteria for participants	✓ Methods
4b	Settings and locations where the data were collected	✓ Methods
4c	How participants were identified and consented	✓ Methods
Interventions:		
5	The interventions for each group with sufficient details to allow replication, including how and when they were actually administered	✓ Methods
Outcomes:		
6a	Completely defined prespecified assessments or measurements to address each pilot trial objective specified in 2b, including how and when they were assessed	✓ Methods
6b	Any changes to pilot trial assessments or measurements after the pilot trial commenced, with reasons	N/A
6c	If applicable, prespecified criteria used to judge whether, or how, to proceed with future definitive trial	N/A
Sample size:		
7a	Rationale for numbers in the pilot trial	✓ Methods
7b	When applicable, explanation of any interim analyses and stopping guidelines	N/A
Randomisation:		
Sequence generation:		
8a	Method used to generate the random allocation sequence	N/A
8b	Type of randomisation(s); details of any restriction (such as blocking and block size)	N/A
Allocation concealment mechanism:		
9	Mechanism used to implement the random allocation sequence (such as sequentially numbered containers), describing any steps taken to conceal the sequence until interventions were assigned	N/A
Implementation:		
10	Who generated the random allocation sequence, enrolled participants, and assigned participants to interventions	N/A
Blinding:		
11a	If done, who was blinded after assignment to interventions (eg, participants, care providers, those assessing outcomes) and how	N/A
Analytical methods:		
12a	Methods used to address each pilot trial objective whether qualitative or quantitative	✓ Methods
Results		
Participant flow (a diagram is strongly recommended):		
13a	For each group, the numbers of participants who were approached and/or assessed for eligibility, randomly assigned, received intended treatment, and were assessed for each objective	✓ Results
13b	For each group, losses and exclusions after randomisation, together with reasons	N/A
Recruitment:		
14a	Dates defining the periods of recruitment and follow-up	✓ Results
14b	Why the pilot trial ended or was stopped	N/A
Baseline data:		
15	A table showing baseline demographic and clinical characteristics for each group	✓ Results
Numbers analysed:		
16	For each objective, number of participants (denominator) included in each analysis. If relevant, these numbers should be by randomised group	✓ Results
Outcomes and estimation:		
17a	For each objective, results including expressions of uncertainty (such as 95% confidence interval) for any estimates. If relevant, these results should be by randomised group	✓ Results
Ancillary analyses:		
18	Results of any other analyses performed that could be used to inform the future definitive trial	✓ Results
Harms:		
19	All important harms or unintended effects in each group (for specific guidance see CONSORT for harms)	N/A
19a	If relevant, other important unintended consequences	N/A
Discussion		
Limitations:		
20	Pilot trial limitations, addressing sources of potential bias and remaining uncertainty about feasibility	✓ Discussion
Generalisability:		
21	Generalisability (applicability) of pilot trial methods and findings to future definitive trial and other studies	✓ Discussion
Interpretation:		
22	Interpretation consistent with pilot trial objectives and findings, balancing potential benefits and harms, and considering other relevant evidence	✓ Discussion
22a	Implications for progression from pilot to future definitive trial, including any proposed amendments	✓ Discussion
Other information		
Registration:		
23	Registration number for pilot trial and name of trial registry	✓ Title page
Protocol:		
24	Where the pilot trial protocol can be accessed, if available	✓ Methods
Funding:		
25	Sources of funding and other support (such as supply of drugs), role of funders	✓ Discussion
26	Ethical approval or approval by research review committee, confirmed with reference number	✓ Methods

## Appendix 2

### A list of surgical procedures which were excluded from the survey distribution list

**Table:**

Item	Description	Item	Description	Item	Description
11,333	Caloric test of labyrin	16,633	PROCEDURE ON MULTIPLE PRE	49,342	HIP REVISION TOTAL REPLAC
11,336	Simultaneous bithermal ca	16,636	Procedure on multiple pre	49,345	HIP REVISION TOTAL REPLAC
11,339	Electronystagmography	18,262	ILIO-INGUINAL ILIOHYPOGAS	49,346	HIP REVISION ARTHROPLASTY
114	Removal of calculus. fir	30,569	ENDOSCOPIC EXAMINATION OF	49,515	KNEE REMOVAL OF PROSTHESI
115	Removal of calculus. sub	32,030	RECTOSIGMOIDECTOMY—HART	49,517	KNEE HEMIARTHROPLASTY OF
11,500	Bronchospirometry	32,072	SIGMOIDOSCOPIC EXAMINATIO	49,518	KNEE TOTAL REPLACEMENT AR
11,503	Measurement of: (a) the m	32,075	SIGMOIDOSCOPIC EXAMINATIO	49,519	KNEE TOTAL REPLACEMENT AR
11,505	Measurement of spirometry	32,078	SIGMOIDOSCOPIC EXAMINATIO	49,521	KNEE TOTAL REPLACEMENT AR
11,506	Measurement of respirator	32,081	SIGMOIDOSCOPIC EXAMINATIO	49,524	KNEE TOTAL REPLACEMENT AR
11,507	Measurement of spirometry	32,084	Sigmoidoscopy or colonoscopy	49,527	KNEE TOTAL REPLACEMENT AR
11,508	Maximal symptom‑lim	32,087	Endoscopic examination of	49,530	KNEE TOTAL REPLACEMENT AR
11,600	BLOOD PRESSURE MONITORING	32,088	Fibreoptic colonoscopy ex	49,533	KNEE TOTAL REPLACEMENT AR
11,602	Investigation of venous r	32,089	Endoscopic examination of	49,534	KNEE PATELLO-FEMORAL JOIN
11,604	Plethysmographic assessment	32,090	FIBREOPTIC COLONOSCOPY -	49,536	KNEE REPAIR OR RECONSTRUC
11,605	Infrared photoplethysmogr	32,093	FIBREOPTIC COLONOSCOPY -	49,539	KNEE RECONSTRUCTIVE SURGE
11,610	Measurement of ankle	32,095	ENDOSCOPIC EXAMINATION OF	49,542	KNEE RECONSTRUCTIVE SURGE
11,611	Measurement of wrist	32,222	Endoscopic examination of	49,545	KNEE REVISION ARTHRODESIS
11,612	Exercise study	32222P	Endoscopic exam of the co	49,548	KNEE REVISION OF PATELLO-
11,722	Implanted ECG loop record	32,223	Colonoscopy Applicable On	49,551	KNEE REVISION OF PROCEDUR
11,728	Implanted loop recording	32223P	Col with Polyp Applicable	49,554	KNEE REVISION OF TOTAL RE
11,820	Capsule endoscopy	32,224	Endoscopic examination of	49,557	KNEE DIAGNOSTIC ARTHROSCO
12,210	Overnight paediatric investigation	32224P	Endoscopic exam of the co	50,353	HIP SPICA INITIAL APPLICA
12,213	Overnight paediatric investigation	32,225	Endoscopic examination of	50,616	SCOLIOSIS IN A CHILD OR A
12,215	Overnight paediatric investigation	32225P	Endoscopic exam of the co	50,620	SCOLIOSIS IN A CHILD OR A
12,217	Overnight paediatric investigation	32,226	Endoscopic examination of	50,624	SCOLIOSIS IN A CHILD OR A
13,200	Assisted reproductive ser	32226P	Endoscopic exam of the co	50,628	SCOLIOSIS IN A CHILD OR A
13,203	Ovulation monitoring serv	32,227	Endoscopic examination of	50,636	SCOLIOSIS IN A CHILD OR A
13,206	Assisted reproductive ser	32,228	Colonoscopy Applicable On	50,640	SCOLIOSIS IN A CHILD OR A
13,209	Planning and management o	32228P	Colonoscopy with polyp Ap	55,118	HEART 2 DIMENSIONAL REAL
13,212	OOCYTE RETRIEVAL BY ANY M	35,643	EVACUATION OF THE CONTENT	55,700	Pelvis or abdomen, pregnancy
13,215	TRANSFER OF EMBRYOS OR BO	35,674	ULTRASOUND GUIDED NEEDLIN	55,703	Pelvis or abdomen, pregnancy
13,218	PREPARATION AND TRANSFER	35,676	ECTOPIC PREGNANCY REMOVAL	55,704	Pelvis or abdomen, pregnancy
13,221	Preparation of semen for	35,677	ECTOPIC PREGNANCY REMOVAL	55,705	Pelvis or abdomen, pregnancy
13,251	Intracytoplasmic sperm in	35,678	ECTOPIC PREGNANCY LAPAROS	55,706	Pelvis or abdomen, pregnancy
135A	Consultant paediatrician,	36,504	RIGID CYSTOSCOPY using bl	55,707	Pelvis or abdomen, pregnancy
164A	Professional attendance f	36,505	RIGID CYSTOSCOPY using bl	55,708	Pelvis or abdomen, pregnancy
16,500	Antenatal attendance	36,507	RIGID CYSTOSCOPY using bl	55,709	Pelvis or abdomen, pregnancy
16,501	External cephalic version	36,508	RIGID CYSTOSCOPY using bl	55,712	Pelvis or abdomen, pregnancy
16,502	Polyhydramnios, unstable	36,812	CYSTOSCOPY WITH URETHROSC	55,715	Pelvis or abdomen, pregnancy
16,504	Treatment of habitual mis	36,860	ENDOSCOPIC EXAMINATION OF	55,718	Pelvis or abdomen, pregnancy
16,505	Threatened abortion	38,218	SELECTIVE CORONARY ANGIOG	55,721	Pelvis or abdomen, pregnancy
16,508	Pregnancy complicated by	38,285	IMPLANTABLE EGG LOOP RECO	55,723	Pelvis or abdomen, pregnancy
16,509	Preeclampsia, eclampsia o	38,286	IMPLANTABLE EGG LOOP RECO	55,725	Pelvis or abdomen, pregnancy
16,511	CERVIX PURSE STRING LIGAT	38,288	Implantable loop recorder	55,759	Pelvis or abdomen, pregnancy
16,512	CERVIX REMOVAL OF PURSE S	47,540	HIP SPICA OR SHOULDER SPI	55,762	pelvis or abdomen, pregnancy
16,514	Antenatal cardiotocograph	48,912	SHOULDER ARTHROTOMY OF AN	55,764	pelvis or abdomen, pregnancy
16,515	MANAGEMENT OF VAGINAL DEL	48,915	SHOULDER HEMI-ARTHROPLAST	55,766	Pelvis or abdomen, pregnancy
16,518	Management of labour	48,918	SHOULDER TOTAL REPLACEMEN	55,768	Pelvis or abdomen, pregnancy
16,519	MANAGEMENT OF LABOUR AND	48,921	SHOULDER TOTAL REPLACEMEN	55,770	pelvis or abdomen, pregnancy
16519L	MANAGEMENT OF LABOUR AND	48,924	SHOULDER TOTAL REPLACEMEN	55,772	Pelvis or abdomen, pregnancy
16,520	CAESAREAN SECTION AND POS	48,927	SHOULDER PROSTHESIS REMOV	55,774	Pelvis or abdomen, pregnancy
16,522	MANAGEMENT OF LABOUR AND	48,933	SHOULDER STABILISATION PR	55,816	Hip or groin, 1 or both s
16,525	Management of second trim	48,936	SHOULDER SYNOVECTOMY OF A	55,818	Hip or groin, 1 or both s
16,530	Management of pregnancy l	48,939	SHOULDER ARTHRODESIS OF A	55,820	Paediatric hip examination
16,531	Management of pregnancy l	48,942	SHOULDER ARTHRODESIS OF I	55,822	Paediatric hip examination
16,564	EVACUATION OF RETAINED PR	48,954	SHOULDER ARTHROSCOPIC TOT	55,852	Paediatric spine, spinal
16,567	MANAGEMENT OF POSTPARTUM	48,960	SHOULDER RECONSTRUCTION O	55,854	Paediatric spine, spinal
16,570	ACUTE INVERSION OF THE UT	49,303	HIP ARTHROTOMY OF INCLUDI	57,522	Knee NR K
16,571	CERVIX REPAIR OF EXTENSIV	49,306	HIP—ARTHRODESIS OF ANAE	57,523	Knee R K
16,573	THIRD DEGREE TEAR INVOLVI	49,309	HIP ARTHRECTOMY OR EXCISI	57,537	Knee NR NK
16,590	Planning and management o	49,312	HIP ARTHRECTOMY OR EXCISI	57,540	Knee R NK
16,600	Amniocentesis, diagnostic	49,315	HIP ARTHROPLASTY OF UNIPO	57,700	Shoulder or scapula (NR
16,603	Chorionic villus sampling	49,318	HIP TOTAL REPLACEMENT ART	57,703	Shoulder or scapula (R)
16,606	FETAL BLOOD SAMPLING USIN	49,319	HIP TOTAL REPLACEMENT OF	57,712	Hip joint (R)
16,609	FETAL INTRAVASCULAR BLOOD	49,321	HIP TOTAL REPLACEMENT ART	66,750	Quantitation, in pregnancy
16,612	FETAL INTRAPERITONEAL BLO	49,324	HIP TOTAL REPLACEMENT ART	66,751	Quantitation, in pregnancy
16,615	FETAL INTRAPERITONEAL BLO	49,327	HIP TOTAL REPLACEMENT ART	73,806	Pregnancy test by 1 or more
16,618	AMNIOCENTESIS THERAPEUTIC	49,330	HIP TOTAL REPLACEMENT ART	D10009	Paediatric Dentistry
16,621	AMNIOINFUSION FOR DIAGNOS	49,333	HIP TOTAL REPLACEMENT ART	T049	Procedure Only Paediatric
16,624	FETAL FLUID FILLED CAVITY	49,336	HIP TREATMENT OF A FRACTU		
16,627	FETO-AMNIOTIC SHUNT INSER	49,339	HIP REVISION TOTAL REPLAC		

## Appendix 4

### COVID-19 response during pilot data collection

The last two weeks of pilot data collection was impacted at the two sites in New South Wales due to a wave of COVID-19 community infections. Due to this, there was an amendment to the study protocol. The amendment included the addition of a “*COVID-19 contingency plan*”. The following explanation was provided to the Human Research Ethics Committee: Due to the global COVID-19 pandemic, states and territories within Australia may experience a “lockdown” over a period of time following a COVID-19 outbreak. Pending on the state or territory, and the specific government rules and regulations in place at the time, elective surgery across public and private health may be impacted. The following risk based approach will guide the patient survey distribution for the AusPROM study over such periods of lockdown: “During period of COVID-19 lockdown, hospitals will be managed on a state-by-state basis. Hospitals will be categorised as Green / Amber / Red pending on the specific COVID-19 lockdown rules and regulations in place at the time. A detailed log will be kept to reflect any occasions of lockdown and the categorisation in place.”

- Green
  - No change to surveys 1 (pre-surgery), 2 (post-surgery) or 3 (post-surgery)
- Amber
  - No change to surveys 2 (post-surgery) and 3 (post-surgery), however 1 (pre-surgery) will be managed differently
  - Survey 1 (pre-surgery) will undergo a process agreed to by the General Manger and the Director of Nursing, together with the National Manager Patient Reported Experience and Outcomes
    - Pending on the situation, it is likely that this will involve a process of automating a list of patients who have confirmed surgery on a more frequent basis, to account for the likelihood of cancellations
    - This many include a more conservative approach to survey distribution, where patients with a “likelihood” of having surgery cancelled, being removed from the Survey 1 distribution list (noting they will be picked up for Survey 2 and 3 if they do in fact have the elective surgery)
- Red
  - No change to surveys 2 (post-surgery) and 3 (post-surgery), however survey 1 (pre-surgery) will be stopped due to the human demands associated with the party automated / party manual process

As such, due to the COVID-19 pandemic, the two sites in NSW entered a “Red Phase” during the final 2 weeks of data collection. This involved no change to surveys 2 (post-surgery) or 3 (post-surgery), however survey 1 (pre-surgery) was stopped due to the human demands associated with the party automated/party manual process.
